# MRI characterisation of adult onset alpha-methylacyl-coA racemase deficiency diagnosed by exome sequencing

**DOI:** 10.1186/1750-1172-8-1

**Published:** 2013-01-03

**Authors:** Kristoffer Haugarvoll, Stefan Johansson, Charalampos Tzoulis, Bjørn Ivar Haukanes, Cecilie Bredrup, Gesche Neckelmann, Helge Boman, Per Morten Knappskog, Laurence A Bindoff

**Affiliations:** 1 Department of Neurology, Haukeland University Hospital, Bergen, Norway; 2 Department of Clinical Medicine, University of Bergen, Bergen, Norway; 3 Center for Medical Genetics and Molecular Medicine, Haukeland University Hospital, Bergen, Norway; 4 Department of Biomedicine, University of Bergen, Bergen, Norway; 5 Department of Ophthalmology, Haukeland University Hospital, Bergen, Norway; 6 Department of Radiology, Haukeland University Hospital, Bergen, Norway

**Keywords:** *AMACR* gene, Seizures, Next generation sequencing, Ataxia, Peroxisomal disorders, Metabolic disorders, Tremor, Peripheral neuropathy, Pigmentary retinopathy

## Abstract

**Background:**

Correct diagnosis is pivotal to understand and treat neurological disease. Herein, we report the diagnostic work-up utilizing exome sequencing and the characterization of clinical features and brain MRI in two siblings with a complex, adult-onset phenotype; including peripheral neuropathy, epilepsy, relapsing encephalopathy, bilateral thalamic lesions, type 2 diabetes mellitus, cataract, pigmentary retinopathy and tremor.

**Methods:**

We applied clinical and genealogical investigations, homozygosity mapping and exome sequencing to establish the diagnosis and MRI to characterize the cerebral lesions.

**Results:**

A recessive genetic defect was suspected in two siblings of healthy, but consanguineous parents. Homozygosity mapping revealed three shared homozygous regions and exome sequencing, revealed a novel homozygous c.367 G>A [p.Asp123Asn] mutation in the *α-methylacyl-coA racemase (AMACR)* gene in both patients. The genetic diagnosis of α-methylacyl-coA racemase deficiency was confirmed by demonstrating markedly increased pristanic acid levels in blood (169 μmol/L, normal <1.5 μmol/L). MRI studies showed characteristic degeneration of cerebellar afferents and efferents, including the dentatothalamic tract and thalamic lesions in both patients.

**Conclusions:**

Metabolic diseases presenting late are diagnostically challenging. We show that appropriately applied, homozygosity mapping and exome sequencing can be decisive for establishing diagnoses such as late onset α-methylacyl-coA racemase deficiency, an autosomal recessive peroxisomal disorder with accumulation of pristanic acid. Our study also highlights radiological features that may assist in diagnosis. Early diagnosis is important as patients with this disorder may benefit from restricted dietary phytanic and pristanic acid intake.

## Background

High-throughput sequence capture methods and next generation sequencing (NGS) technologies make exome sequencing an effective method to identify the cause of Mendelian disorders [1-3]. Exome sequencing has primarily been applied to identify novel genetic causes of disease although it is increasingly being used in the diagnostic setting, particular in disorders displaying locus heterogeneity such as ataxias, Charcot-Marie-Tooth disease (CMT), hereditary spastic paraplegias (HSP) and retinitis pigmentosa [3-5]. Large genes containing numerous mutations may also be effectively screened by NGS. In addition, exome sequencing may be an important tool for correctly diagnosing rare disorders, [6] particularly when they manifest phenotypic heterogeneity.

We used exome sequencing to diagnose α-methylacyl-coA racemase deficiency (MIM 614307), an autosomal recessive peroxisomal disorder with accumulation of pristanic acid, in two adult siblings. α-methylacyl-coA racemase deficiency has previously been reported in eight adult patients worldwide and the disorder has been associated with heterogeneous clinical presentations (Table 1) [7-14]. Peroxisomes are involved in the breakdown of very-long-chain fatty acids (VLCFAs), polyunsaturated fatty acids, and branched-chain fatty acids like phytanic and pristanic acid. In addition, peroxisomes are involved in the formation of primary C24-bile acids by β-oxidation of the bile-acid intermediates di- and trihydroxycholestanoic acids (DHCA and THCA) [15]. In peroxisomal disorders affecting breakdown of branched-chain fatty acids, phytanic acid and pristanic acid accumulate [16]. Phytanic acid and pristanic acid are degradation products from the chlorophyll side-chain phytol. Both phytanic acid and pristanic acid are derived directly from dietary sources such as meat and dairy products from ruminants. Additionally, oils, fats and milk derived from molluscs, fish and whales are important sources of these fatty acids in humans [17]. Phytanic acid is metabolized via peroxisomal α-oxidation to pristanic acid. Pristanic acid undergoes β-oxidation in peroxisomes with subsequent β-oxidation in mitochondria. Peroxisomal biogenesis disorders (PBD), e.g. Zellweger syndrome (MIM 214100), are characterized by defects in both α- and β-oxidation. In contrast, single enzyme deficiencies (SED) have either deficient α- or β-oxidation [18]. Peroxisomal SED include X-linked adrenoleukodystrophy (MIM 300100) , Refsum disease (MIM 266500), α-methylacyl-coA racemase (AMACR) deficiency (MIM 614307), sterol carrier protein X (SCPx) deficiency (MIM 613724) and D-bifunctional protein (DBP) deficiency (MIM 261515) [9,15,19].

**Table 1:** Clinical features in adult α-methylacyl-coA racemace deficiency patients

	**Pt 1 **[9,11]	**Pt 2 **[9]	**Pt 3 **[7]	**Pt 4 **[13]	**Pt 5 **[12]	**Pt 6 **[10]	**Pt 7 **[8]	**Pt 8 **[14]	Pt A*	Pt B*
Age of onset/Gender	18/M	48/F	36/F	13/F	2^nd^ decade/ M	3^rd^ decade/ M	50/M	3^nd^ decade/M	30/M	33/F
Presentation	developmental delay, episode with blindness	spastic paraparesis	tremor	seizures	seizures	neuroleptic malignant syndrome with rhabdomyolysis	gait ataxia, dysarthria	seizures	arm weakness	seizures
Seizures	Yes	No	Yes	Yes	Yes	Yes	Yes	Yes	Yes	Yes
Encephalopathic episode(s)	Yes	No	Yes	Yes	Yes	Yes	No	Yes	Yes	Yes
Tremor	No	No	upper limbs, head, voice	No	No	No	No	No	No	upper limbs, head
Cerebellar	No	No	dysarthria	No	No	No	gait ataxia, dysarthria	No	No	No
Neuropathy	sensory-motor	sensory-motor	No	mild sensory	mononeu-ropathies	sensory axonal	sensory-motor		sensory-motor	sensory-motor
Retinopathy	RP	N/A	RP	No	RP	degenera-tive retino-pathy	N/A	pigmentary retinopathy	pigmentary retinopathy	pigmentary retinopathy
DM II	N/A	N/A	N/A	N/A	N/A	N/A	N/A	No	Yes	Yes
Additional Features	primary hypogonadism, migraine	migraine	cataract, migraine, hyperreflexia depression	cognitive decline, unsteady gait, depression	primary hypogonadism	schizophrenia,	decline in short-term memory	low s-testosterone	cataract	cataract
*AMACR*-gene mutation	c.154 T>C [p.Ser52Pro]	c.154 T>C [p.Ser52Pro]	c.154 T>C [p.Ser52Pro]	c.154 T>C [p.Ser52Pro]	c.154 T>C [p.Ser52Pro]	c.559 G>A [p.Gly187Arg]	c.154 T>C [p.Ser52Pro]	N/A	c.367 G>A, [p.Asp123Asn]	c.367 G>A, [p.Asp123Asn]

Summary of reported clinical features in adult α-methylacyl-coA racemace deficiency patients. Pt=patient, RP=retinitis pigmentosa, DM II= type 2 diabetes mellitus.

**Figure 1:**
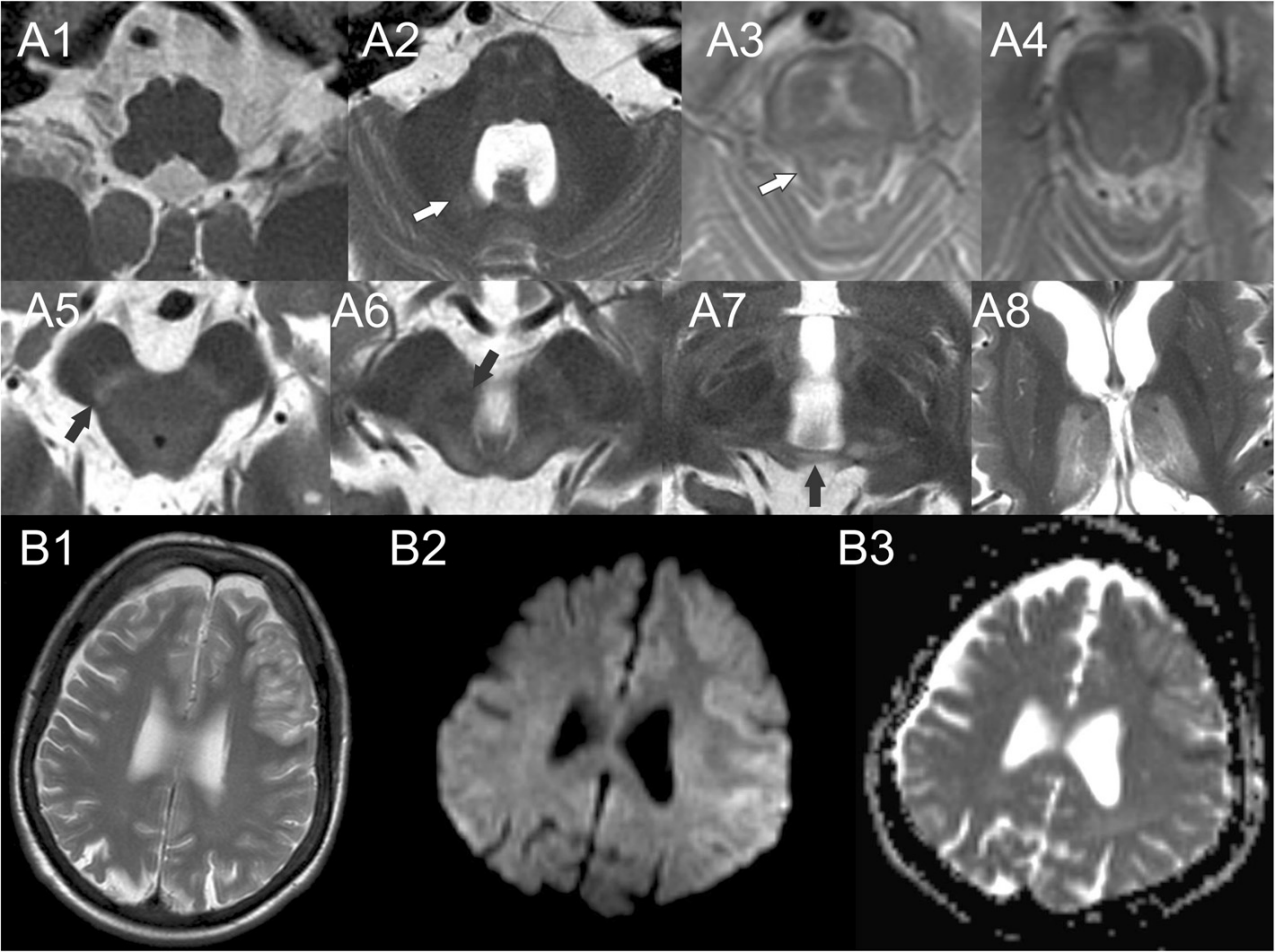
**Chronic and acute brain MRI findings in α-methylacyl-coA racemace deficiency. A)** Representative axial T_2_-images showing different brainstem levels and the thalami in a caudocranial direction. There is evidence of chronic degeneration of both cerebellar efferent and afferent pathways. **A1)** No evidence of inferior olivary lesion in the medulla oblongata. **A2)** High signal of the dentate nucleus (arrow). This finding was preceded by loss of T_2_ hypointensity. High signal and atrophy of the pons is also apparent at this level. **A3)** High signal in the superior cerebellar peduncle (SCP; arrow). In addition, there is degeneration of the cerebellar afferents with pontine atrophy and hyperintensity corresponding to the transverse pontine fibers. **A4)** High signal corresponding to the decussation of SCP. **A5)** High signal corresponding to the dentatothalamic tract (arrow) at the level of the inferior colliculus in the midbrain. **A6)** Probable loss of T_2_ hypointensity in the red nucleus (arrow) on 3 Tesla MRI and high signal in the superior colliculus. **A7)** High signal in the posterior commissure (arrow). **A8)** High signal in the thalami. **B)** Acute lesion two weeks into an encephalopatic episode with aphasia, right upper limb paresis, central facial palsy and reduced consciousness in patient B. Left frontal high T_2_-signal cortical lesion **(B1)**, showing a mixed diffusion pattern with areas of both high and low apparent diffusion coefficient (ADC) (B2-B3).

We report two siblings with a novel homozygous missense mutation (c.367 G>A; [p.Asp123Asn]) in the *AMACR-*gene. The diagnosis of α-methylacyl-coA racemase deficiency was made using exome sequencing and confirmed by demonstrating markedly increased pristanic acid levels in blood. Furthermore, we provide a detailed description of the specific MRI findings in this disorder (Figure 1). Our findings highlight the neuroanatomical structures affected in α-methylacyl-coA racemase deficiency and show that MRI has high diagnostic specificity early in the course of the disease.

## Methods

### Samples

Two siblings of healthy, consanguineous parents with similar clinical and radiological findings were examined on several occasions during the course of their disease. Our study was approved by the Regional Committee for Medical and Health Research Ethics, Western Norway (IRB00001872). All study participants provided written informed consent.

### Ophthalmological investigation

Patient B underwent a thorough clinical ophthalmological examination including determination of colour vision by Farnsworth D15, visual field by Goldmann perimetry and retinal thickness and structure by Optical coherence tomography. Fullfield electroretinogram was recorded as previously described [20]. Patient A had undergone several ophthalmological examinations and his medical records were reviewed for this study.

### Genetic studies

Genealogical data from the patients, local parish registers and population censuses were used. Whole genome, single nucleotide polymorphism (SNP) genotyping was performed utilizing the Genome-Wide Human SNP Array 6.0 (Affymetrix, Santa Clara, CA) and homozygosity mapping performed using the PLINK program [21]. Targeted capture and exome sequencing were performed at HudsonAlpha Institute for Biotechnology (Huntsville, AL); Exome capture was performed using Roche-NimbleGen Sequence Capture EZ Exome v2 kit and paired-end 100nt sequencing on the Illumina HiSeq. The paired-end reads were analyzed with Casava v1.8 (Illumina Inc) and aligned to the hg19 reference genome using Burrows-Wheeler Alignment tool (BWA) [22]. This resulted in 11.0 Giga-bases of aligned sequence data and 124X median coverage of the target capture regions. PCR duplicates were removed with PICARD (http://picard.sourceforge.net) and the Genome analysis toolkit (GATK) was used for base quality recalibration [23]. SNPs and indels were called by SAMtools using a minimum threshold of 8X sequencing depth and quality score ≥ 30 [24]. Annovar was used for variant annotation [25]. Variant prioritization was performed based on a recessive model, filtering against variants identified in more than 80 Norwegian exome-resequencing samples (obtained using the same whole exome sequencing pipe-line) and variants present at >0.5% allele frequency in the 1000 Genomes database (phase 1 project data 2010.08.04 sequence index, (26.1 million SNPs released in November 2010 and 3.7 million indels released in February 2011). Variants not in the CCDS or in putative splice sites (defined as 2 bps flanking coding exons) and synonymous variants were also excluded.

### Radiological investigations

Three brain MRI studies from patient A and nine from patient B were available for investigation. The MRI scans had been acquired over three and 11 years, respectively and this sequential data allowed us to study lesion evolution both during the chronic and acute phases of the disease. Diffusion imaging was performed during acute encephalopathy episodes. MRI was performed on a Siemens Magnetom Symphony 1.5 T scanner with 30 mT/m gradients or a General Electric Signa Excite 3 T HDX scanner with 40 mT/m gradients. Sequences included T1, T2 and T2 fluid-attenuated inversion recovery. Diffusion imaging was performed with b values of 0, 500 and 1000.

Measurement of blood pristanic acid level was performed using a standard diagnostic protocol.

## Results

### Clinical characteristics

#### Patient A

At age 30 years this man reported a feeling of weakness in his upper limbs. Two years later he developed periodic discomfort in his lower extremities. Subsequently, he experienced fatigue following moderate physical activity and episodic elevation of serum creatine kinase (CK) and lactate dehydrogenase (LDH) were found. He underwent a brief ophthalmic examination at the age of 37 at which time bilateral incipient cataracts were noted. No retinal degeneration was described. At age 39 years, a demyelinating peripheral neuropathy was diagnosed and a glucose tolerance test was consistent with type 2 diabetes mellitus. At this time, his liver enzymes were mildly increased and ultrasound of the liver showed steatosis. At age 48 years he developed an acute encephalopathic episode with headache and profound bilateral visual loss (no perception of light). Ophthalmoscopy revealed normal optic discs with a bilateral pigmentary retinopathy most pronounced in the mid-periphery but also involving the macula. Visual evoked potential suggested post-chiasmal damage. Thirteen days after onset of the episode he developed complex partial seizures with rotation of the head, repetitive hand movements in his right upper limb and reduced conciseness. His visual acuity gradually improved over the next year to 0.32 (Snellen) in both eyes. The lens opacities remained stable and cataract surgery was not indicated. At age 51 years a liver tumor was diagnosed, biopsy showed probable liver-sarcoma. He died within months and autopsy was declined.

#### Patient B

The patient is the sister of patient A and she developed her first symptoms age 33 years during pregnancy. At this time she experienced several episodes of dysarthria combined with numbness and impaired control in her left arm, lasting for a few minutes. These were probably partial seizures and she had two further episodes age 40, but the diagnosis of epilepsy was not established until she was 49 years old and had several episodes of dysphasia, right sided central facial palsy and upper right limb numbness. Type 2 diabetes mellitus was diagnosed aged 48 years. In her late forties she gradually developed upper limb tremor, with both positional and intentional characteristics, and later head tremor; the tremor is alcohol responsive. Peripheral neuropathy was diagnosed age 57. At age 53 years she had an acute encephalopathic episode associated with aphasia, weakness of her right arm and upper motor neuron facial palsy that persisted for days and with episodic worsening. MRI showed left sided frontotemporal cortical edema (Figure 1). Following admission, she had several complex partial seizures with right sided facial twitching and rotation of the head to the right side. She improved with lamotrigine treatment. The patient has been started on a diet to restrict phytanic and pristanic acid intake, however it is too early to evaluate any benefit.

#### Ophthalmological findings

**Figure 2:**
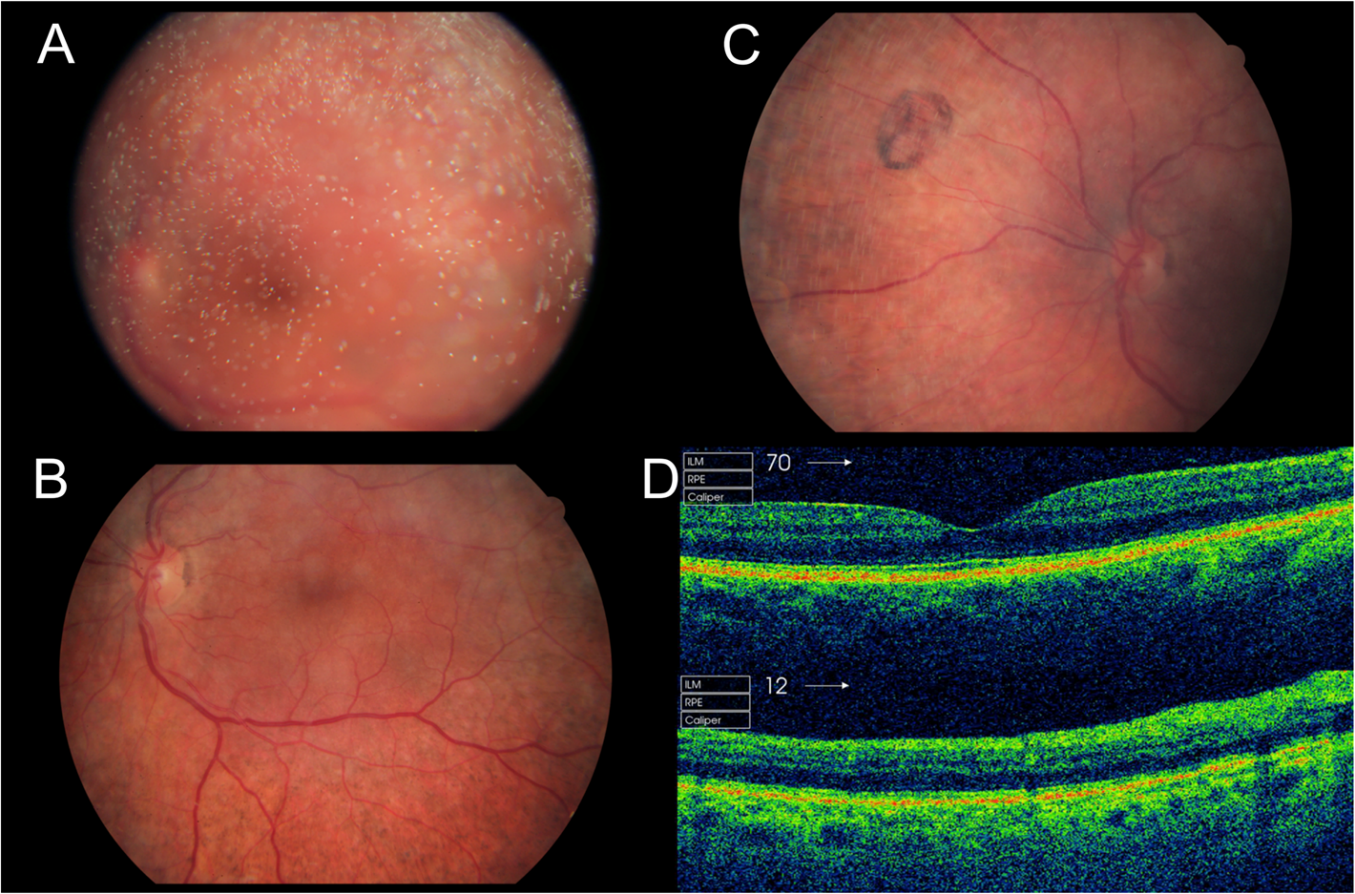
**Ophthalmic features in patient B. A – C)** Fundus images of the right eye showing pronounced asteroid hyalosis **(A)**. Pigment epithelial atrophy and moderate pigmentary retinopathy are present in the mid-periphery. The macula, optic disc and retinal vessles are normal **(B)**. Areas resembling pigmented and nonpigmentet small congenital hypertrophy of the retinal pigment epithelium were present **(C). D)** Optical coherence tomography showing a normal macula (top) but peripheral retinal pigment layer degeneration and disturbances in the photoreceptor layer (bottom).

Patient B underwent bilateral, uncomplicated cataract surgery at the age of 51. She obtained good postoperatively visual acuity but experienced a subjectively delayed adaption to dark. At the age of 59 she had best corrected visual acuity of 0.8 (Snellen) in both eyes. Exophoria and reduced convergence were noted. Slightly prominent corneal nerves and keratopathy of the left eye were found on slit lamp examination. She had pronounced bilateral asteroid hyalosis (Figure 2A). On ophthalmoscopy pigment epithelial atrophy and moderate pigmentary retinopathy was seen in the mid-periphery (Figure 2B-C).

**Figure 3:**
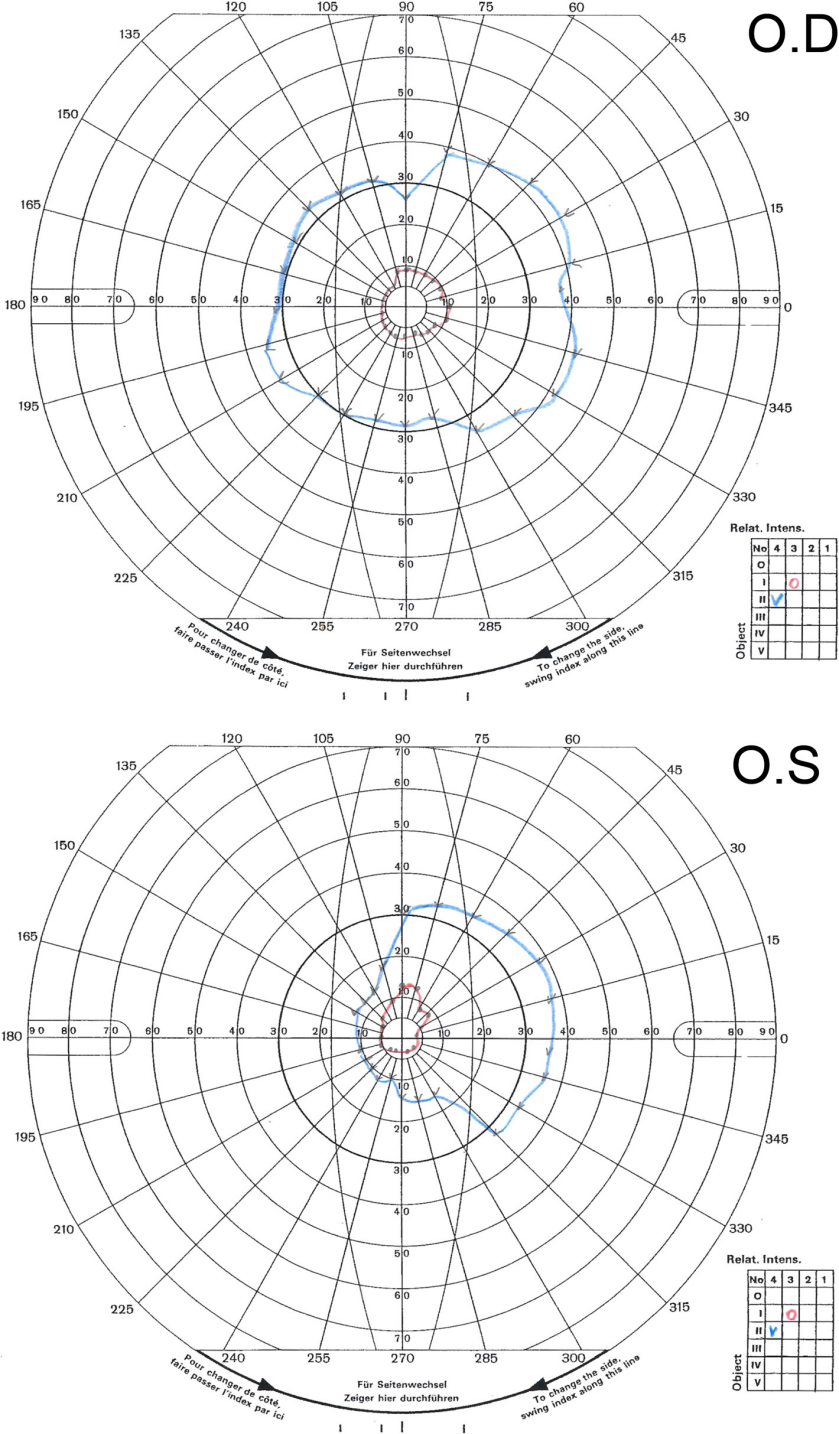
**Goldmann perimetry in patient B.** Constricted visual fields were found. In the right eye a conserved central horizontal field of 30 degrees nasally and 40 degrees temporally are seen. In the left eye the conserved visual field is 10 degrees nasally and 40 degrees temporally.

Constricted visual fields were found on Goldmann perimetry (Figure 3). Colour vision was normal. On optical coherence tomography peripheral retinal pigment layer degeneration and slightly disturbed photoreceptors were noted (Figure 2D). Full-field electroretinogram showed normal to subnormal amplitudes of the isolated rod response, subnormal amplitudes for the combined rod-cone response and normal values for the 30 Hz flicker cone response. The implicit time for the combined rod-cone response was within normal range whereas prolonged values were found for the 30 Hz cone response (Additional file 1). These findings are indicative of photoreceptor damage.

#### Genetic studies

Genealogical investigation revealed that the parents of the two siblings were consanguineous. SNP linkage showed that the two siblings shared three homozygous regions comprising ~17 Mb, ~15 Mb and ~5 Mb. Based on an assumed recessive inheritance and homozygous mutation, we performed whole exome sequencing in patient B. This identified 19,596 genetic variants, of which 220 were not found in 80 other Norwegian exomes and not present at > 0.5% allele frequency in the 1000 Genomes database (Table 2). Analysis of these 220 rare variants revealed 5 genes with homozygous variants and two with compound heterozygous variants consistent with autosomal recessive inheritance. Filtering using whole genome genotyping data from both affected siblings left us with three genes - *PRDM9, DROSHA, and AMACR*. Homozygous *AMACR* gene mutations are known to cause α-methylacyl-coA racemase deficiency, comprising peripheral neuropathy, seizures and relapsing encephalopathy with MRI findings resembling those found in our patients. Sanger sequencing confirmed the homozygous *AMACR* mutation c.367 G>A [p.Asp123Asn] (NCBI Reference sequence NM_014324.5) in both siblings. The diagnosis of α-methylacyl-coA racemase deficiency was confirmed by finding markedly increased pristanic acid levels levels in blood in patient B (169 μmol/L, normal <1.5 μmol/L).

**Table 2:** Exomic variant filtration

**Filter**	**Count**
Exomic variants	19596
- Excluding synonomous variants	9584
- Not in 80 Norwegian Exomes	251
- Not in 1000Genomes (0.5% MAF)	220
Putative Recessive	7 genes
In homozygous regions shared by both siblings	3 genes

Variant filtration of exome sequencing data from Patient B compared with whole genome genotyping data in both affected siblings reveal three genes consistent with a homozygous causal mutation.

#### Radiological findings

Sequential MRI investigations were available from both patients. These showed a specific, degeneration of cerebellar afferents and efferents (Figure 1). Specifically, we found degeneration of the cerebellothalamic tract; including prolongation of the T_2_ signal in the dentate nucleus, atrophy and hyperintense T_2_-signal of the superior cerebellar peduncle (SCP). This pattern of hyperintense T_2_-signal associated with the cerebellothalamic tract can be followed cranially to the decussation of superior cerebellar peduncle. There is probable prolongation of the T_2_ signal (T_2_ hypointensity loss) in the red nucleus. Furthermore, there is hyperintense T_2_-signal at the level of the superior colliculus, the posterior commissure and the thalami indicating a chronic degeneration of both the dentatorubral and the dentatothalamic tracts. These findings were already present in the first MRI studies in both patients, but progressed during the course of disease; with hyperintense T_2_-signal of the SCP becoming evident in the last scan performed in patient A and gradual prolongation of the T_2_-signal in the dentate nucleus, i.e. development from T_2_-loss to hyperintense T_2_-signal. Interestingly, neither of the two patients had inferior olivary hypertrophy (IOH) as seen in similar lesions, when the Guillain-Mollaret triangle is lesioned [26,27]. In addition, there is degeneration of the cerebellar afferents with pontine atrophy and high signal corresponding to the transverse pontine fibers. The acute lesions corresponded to encephalopatic episodes and were marked by cortical edema and patchy restricted diffusion pattern.

## Discussion

We used exome sequencing to diagnose α-methylacyl-coA racemase deficiency in two adult siblings with a complex autosomal recessive, adult-onset neurological syndrome, comprising peripheral neuropathy, epilepsy, relapsing encephalopathy, bilateral thalamic lesions on MRI, type 2 diabetes mellitus, cataract, pigmentary retinopathy and tremor. A combination of homozygosity mapping and filtering of the exome sequencing data reduced the number of candidate genes to three (Table 2). Re-evaluation of the clinical phenotype and MRI images suggested that our patients were likely to have this peroxisomal disorder, resulting in impaired oxidation of pristanic acid. This diagnosis was biochemically validated by finding markedly increased pristanic acid levels in blood. Thus, confirming that the novel, homozygous c.367 G>A [p.Asp123Asn] *AMACR* mutation is pathogenic. Therefore, no further metabolic characterization was performed in our patients. However metabolic profiling can be used to distinguish between different types within the heterogeneous group of peroxisomal diseases (Table 3) [9].

**Table 3:** Metabolic profiles in peroxisomal disease

	**X-linked adrenoleukodystrophy**	**Refsum disease**	**AMACR deficiency**
VLCFAs	↑↑	-	-
Phytanic acid	-	↑↑↑	(↑)
Pristianic acid	-	-	↑↑↑
THCA	-	-	↑↑
DHCH	-	-	↑↑

Metabolic profiles in blood. VLCFAs = very-long-chain fatty acids, DHCA/THCA = di- / tri-hydroxycholestanoic acids; the bile-acid intermediates.

Alpha-methylacyl-coA racemase deficiency presents heterogeneously in adults, with peripheral neuropathy, pigmentary retinopathy, seizures and relapsing encephalopathy being common features (Table 1). While these were present in our patients, other features, including stroke-like lesions, peripheral neuropathy, type 2 diabetes mellitus and thalamic MRI lesions lead us initially to suspect a mitochondrial syndrome. Type 2 diabetes mellitus has not been described in α-methylacyl-coA racemase deficiency and may be a coincidental finding. Tremor was only observed in one of our patients. These symptoms may represent rare features of this disorder. Both of our patients had early cataract. Cataracts have previously been reported in one patient with additional retinitis pigmentosa [7]. Patient B had pigmentary changes most prominent in the mid-periphery while the macula seemed normal. Similar changes, also involving the right macula, were described in her brother. Unfortunately, no fundus photos were available for comparison. A similar retinopathy, however predominantly in the macula, has been reported in one patient [14]. Retinal changes have been reported in five patients and vary from retinal pigmentary epithelial changes to retinitis pigmentosa [7,9,11,12,14].

Alpha-methylacyl-coA racemase deficiency has also been reported in four infants although clinical presentation differed to that seen in adults with abnormal bile acid synthesis, coagulopathy and neonatal cholestasis [9,28,29]. In addition to the variability seen in patients with α-methylacyl-coA racemase deficiency, deficiency of SCPx, a downstream enzyme in the peroxisomal β-oxidation, causes similar clinical and MRI findings with increased pristanic acid in blood [15]. Our study shows that, properly applied, exome sequencing has the potential to improve the diagnostic process.

Exome sequencing was originally used in the discovery of novel genetic causes of disease [1]. It is, however, increasingly being used as a diagnostic tool to replace Sanger sequencing on a gene-by-gene basis in disorders that display genetic heterogeneity. Exome sequencing allows rapid screening of a large number of candidate genes and at a cost which is no longer prohibitive, it may also be a cost effective diagnostic screening tool [4]. Our study confirms the potential of exome sequencing; however, it also highlights the difficulties associated with the finding of a novel mutation. In our patient, the findings allowed us to re-evaluate the clinical and MRI findings and validate the diagnosis by biochemical measurement of pristanic acid levels. While there are limitations to the sensitivity of exome sequencing, including not covering all exons and exon-intron boundaries, inconsistencies in sequencing depth across the exome and that not all monogenic disorders are located in the exome, [3,30] nevertheless exome sequencing allows a far more complete diagnostic evaluation than has previously been feasible.

Our MRI studies showed the combination of acute and chronic changes. Encephalopathic episodes were associated with acute stroke-like lesions and subsequent cortical atrophy. Chronic changes, including high T_2_ signal in the thalami, midbrain and pons (Figure 1), were present from the earliest stages and appear specific for α-methylacyl-coA racemase deficiency. Both cerebellar afferents and efferents are affected and we show that the dentatothalamic tract is also affected (Figure 1). Gradual progression of the chronic lesions of the cerebellar efferents was also seen. The chronic MRI findings we describe have not previously been reported in detail and appear sufficiently suggestive of this disorder to prompt testing of blood pristanic acid levels and the early diagnosis of α-methylacyl-coA racemase deficiency.

Similar MRI findings have been reported in certain mitochondrial syndromes. Long tract signal abnormalities are typically seen in the syndrome of leukoencephalopathy with brainstem and spinal cord involvement and elevated lactate (LBSL). These patients also have spinal cord changes and diffuse periventricular leukoencephalopathy and lack thalamic involvement [31]. Bilateral thalamic changes and stroke-like lesions are typical of polymerase gamma (POLG) encephalopathy, which may also show similar clinical features [26]. With the exception of inferior olivary lesions, however, brainstem lesions are rare in POLG associated encephalopathy. The combination of brainstem and thalamic lesions may occur in Leigh disease, but here the brainstem changes affect primarily gray matter structures such as the substantia nigra, subthalamic and red nuclei and not white matter tracts [32].

The occurrence of movement disorders in patients with α-methylacyl-coA racemase deficiency is difficult to evaluate. One of our patients (B) had a head tremor (no-no), postural and action tremor of the upper limbs, similar to essential tremor. There was no evidence of parkinsonism or dystonia. Tremor involving the upper limbs, head and voice has been reported previously in a patient who also developed dysarthria [7]. An additional patient had gait ataxia and cerebellar dysarthria [8]. One patient with SCPx deficiency had adult onset cervical dystonia with dystonic head tremor, cerebellar ataxia and increased pristanic acid in blood [15]. Thus, it appears that movement disorder, and particularly tremor, may be a part of the disease. These patients may therefore provide important insights into movement disorders.

It is unclear how α-methylacyl-coA racemase deficiency leads to pigmentary retinopathy and neuronal death. Experimental evidence indicate that pristanic acid may cause dramatic Ca^2+^ deregulation, in situ mitochondrial depolarization and induced generation of reactive oxygen species (ROS) [33,34]. Importantly, a correct diagnosis of α-methylacyl-coA racemase deficiency may have important implications for clinical care. Patients with Refsum disease benefit from restricted phytanic acid intake and plasmapheresis [35,36]. The benefit of restricted phytanic and pristanic acid intake in α-methylacyl-coA racemase deficiency patients is not yet established. However, a stable clinical course has been reported in some patients after initiation of such a diet [12]. Furthermore, acute critical illness may lead to increased release of pristanic acid stored in fat tissue, and patients may benefit from plasmapheresis during episodes of acute illness.

## Conclusions

Adult onset α-methylacyl-coA racemase (AMACR) deficiency (MIM 614307) is a rare autosomal recessive peroxisomal single enzyme deficiency disorder with accumulation of pristanic acid. Peripheral neuropathy, pigmentary retinopathy, seizures and relapsing encephalopathy are common clinical features, however, the clinical presentation is heterogeneous and patients may be mislabelled as possible mitochondrial disease. We used exome sequencing to diagnose AMACR-deficiency in two siblings. Furthermore we provide a detailed description of the MRI features that may guide an early diagnosis in these patients. Early diagnosis is pivotal, as these patients may benefit from restricted dietary phytanic and pristanic acid intake.

## Competing interests

The authors declare that they have no competing interests.

## Authors’ contributions

Study conception and design: Dr KH, Dr SJ, Dr CT, Dr HB, Dr PMK, Dr LAB; Analysis and interpretation of data: Dr KH, Dr SJ, Dr CT, Dr BIH, Dr CB, Dr GN, Dr HB, Dr PMK, Dr LAB; Drafting the article or revising it critically for important intellectual content: Dr KH, Dr SJ, Dr CT, Dr BIH, Dr CB, Dr GN, Dr HB, Dr PMK, Dr LAB. All authors have read and approved the final manuscript.

## Supplementary Material

Additional file 1 Electroretinogram (ERG) in patient B. Click here for file
