# Supplementary material for: MRI characterisation of adult onset alpha-methylacyl-coA racemase deficiency diagnosed by exome sequencing
Source: Orphanet J Rare Dis. 2013 Jan 3;8:1. doi: 10.1186/1750-1172-8-1 (PMC3567975; doi:10.1186/1750-1172-8-1)
Supplement: Additional file 1 — Electroretinogram (ERG) in patient B. [file 1750-1172-8-1-S1.pdf]

Supplemental Material

Additional file 1: Electroretinogram (ERG) in patient B

	Dark-Adapted Dim Blue Single Flash Ampl (µV)	Dark-Adapted White Single Flash Ampl (µV)	Dark-Adapted White Single Flash IT (ms)	Dark-Adapted 30-Hz Flickering White Light Ampl (µV)	Dark-Adapted 30-Hz Flickering White Light IT (ms)
Patient RE/LE	58.6/44.3	198/142	46.2/43.2	42.9/26.9	37.5/34.5
Normal values	51-281	219-511	40-56	22-104	26-32.6

RE = right eye, LE = left eye, Ampl = amplitude, IT = implicit time.
